# Degeneracy in the emergence of spike-triggered average of hippocampal pyramidal neurons

**DOI:** 10.1038/s41598-019-57243-8

**Published:** 2020-01-15

**Authors:** Abha Jain, Rishikesh Narayanan

**Affiliations:** 1 Cellular Neurophysiology Laboratory, Molecular Biophysics Unit, Indian Institute of Science, Bangalore, India; 2 Undergraduate program, Indian Institute of Science, Bangalore, India

**Keywords:** Cellular neuroscience, Biophysical models, Neural encoding, Ion channels in the nervous system, Intrinsic excitability

## Abstract

Hippocampal pyramidal neurons are endowed with signature excitability characteristics, exhibit theta-frequency selectivity — manifesting as impedance resonance and as a band-pass structure in the spike-triggered average (STA) — and coincidence detection tuned for gamma-frequency inputs. Are there specific constraints on molecular-scale (ion channel) properties in the concomitant emergence of cellular-scale encoding (feature detection and selectivity) and excitability characteristics? Here, we employed a biophysically-constrained unbiased stochastic search strategy involving thousands of conductance-based models, spanning 11 active ion channels, to assess the concomitant emergence of 14 different electrophysiological measurements. Despite the strong biophysical and physiological constraints, we found models that were similar in terms of their spectral selectivity, operating mode along the integrator-coincidence detection continuum and intrinsic excitability characteristics. The parametric combinations that resulted in these functionally similar models were non-unique with weak pair-wise correlations. Employing virtual knockout of individual ion channels in these functionally similar models, we found a many-to-many relationship between channels and physiological characteristics to mediate this degeneracy, and predicted a dominant role for HCN and transient potassium channels in regulating hippocampal neuronal STA. Our analyses reveals the expression of degeneracy, that results from synergistic interactions among disparate channel components, in the concomitant emergence of neuronal excitability and encoding characteristics.

## Introduction

Degeneracy, the ability of distinct structural components to elicit similar physiology, is ubiquitous in biology, across organisms and across scales^1–8^. Several studies have shown that models with disparate combinations of ion channel conductances can have physiological measurements with values within their experimentally determined ranges^4,6,8–19^. Most of these studies, however, have focused largely on measures of excitability, with the focus primarily on homeostasis. However, neurons are also endowed with specific physiological properties that define the features that they encode, their operating mode (*i.e*., their position along the integrator-coincidence detector (I-CD) continuum), and coincidence detection capabilities^20–26^. Could degeneracy be a substrate for neurons to encode specific characteristics, to be endowed with specific operational characteristics and still maintain their excitability properties within experimental bounds? Could the feature detection/extraction goals of a neuron coexist with its ability to maintain specific levels of excitability? Could disparate combinations of ion channels mediate such co-existence? Is there a dominance hierarchy among different channels in determining a neuron’s operating mode?

To address these questions, we carried out computational analyses that employed hippocampal CA1 pyramidal neuron models as substrates. We used this particular category of neurons, on account of electrophysiological data availability on ion channel kinetics^27–33^, intrinsic excitability measurements^28,29,34–37^ and measurements of their encoding and operational characteristics^25^. The encoding and operational characteristics of these models were quantified using measurements dependent on the spike-triggered average (STA). The STA of a neuron, defined as the average current stimulus that is required to elicit a spike, is a powerful physiological measurement that represents both encoding and excitability properties of a neuron. The STA provides quantitative assessments of intrinsic excitability, spectral selectivity and neuronal operating mode along the integrator-coincidence detector (I-CD) continuum. These quantifications, showing the STA of hippocampal pyramidal neurons to be endowed with theta-frequency (4–10 Hz) selectivity and gamma-range (25–150 Hz) coincidence detection capabilities^23–26^, place tight constraints on channel properties that result in their emergence^20–26^.

In addition to STA measurements, in our analyses, we placed several available electrophysiological constraints on intrinsic excitability characteristics^35,36,38,39^. Specifically, we employed excitability measures based on pulse current injections (*e.g*., input resistance, firing rate) along with measurements that employ input frequency as a variable (*e.g*., impedance amplitude and phase, resonance frequency). These measurements provided us with the opportunity to physiologically constrain model populations, while also enabling us to assess heterogeneity and degeneracy in these populations. The availability of both encoding as well as excitability measurements enabled us to constrain the operating mode of a given neuron model in a physiologically realistic setting (*i.e*. in the central region, rather than at the more abstract ends of the I-CD continuum^20^) as well as study the concomitant constraints on neuronal excitability. In addition to these physiological constraints on measurements at the cellular scale (neuronal encoding and excitability), we placed explicit biophysical constraints on our models at the molecular scale (ion channels and their properties) based on electrophysiological recordings. We demonstrate, using an unbiased stochastic search algorithm involving thousands of models that yielded biophysically constrained and physiologically validated models, that neurons with disparate channel conductances can concomitantly possess similar encoding and excitability characteristics.

Additionally, we determined the manner in which different ion channels influence various parameters of a neuron’s STA by generating acute virtual knockouts^9,14,40^ of individual ion channels, and by assessing the STA of each virtual knockout model (VKM). We demonstrate that both frequency selectivity and coincidence detection measured from neuronal STA are critically reliant on several ion channels, implying that strong interactions among different ion channels drive operational characteristics and excitability homeostasis in hippocampal pyramidal neurons. These analyses also showed that HCN and transient K^+^ channels are the predominant intrinsic regulators of the hippocampal pyramidal neuron’s operating mode. Our results provide experimentally well-constrained lines of evidence for the expression of degeneracy in the ability of single neurons to be endowed with similar encoding and excitability characteristics, and in determining their operational mode along the I-CD continuum.

## Results

### Base model and its measurements

**Figure 1:**
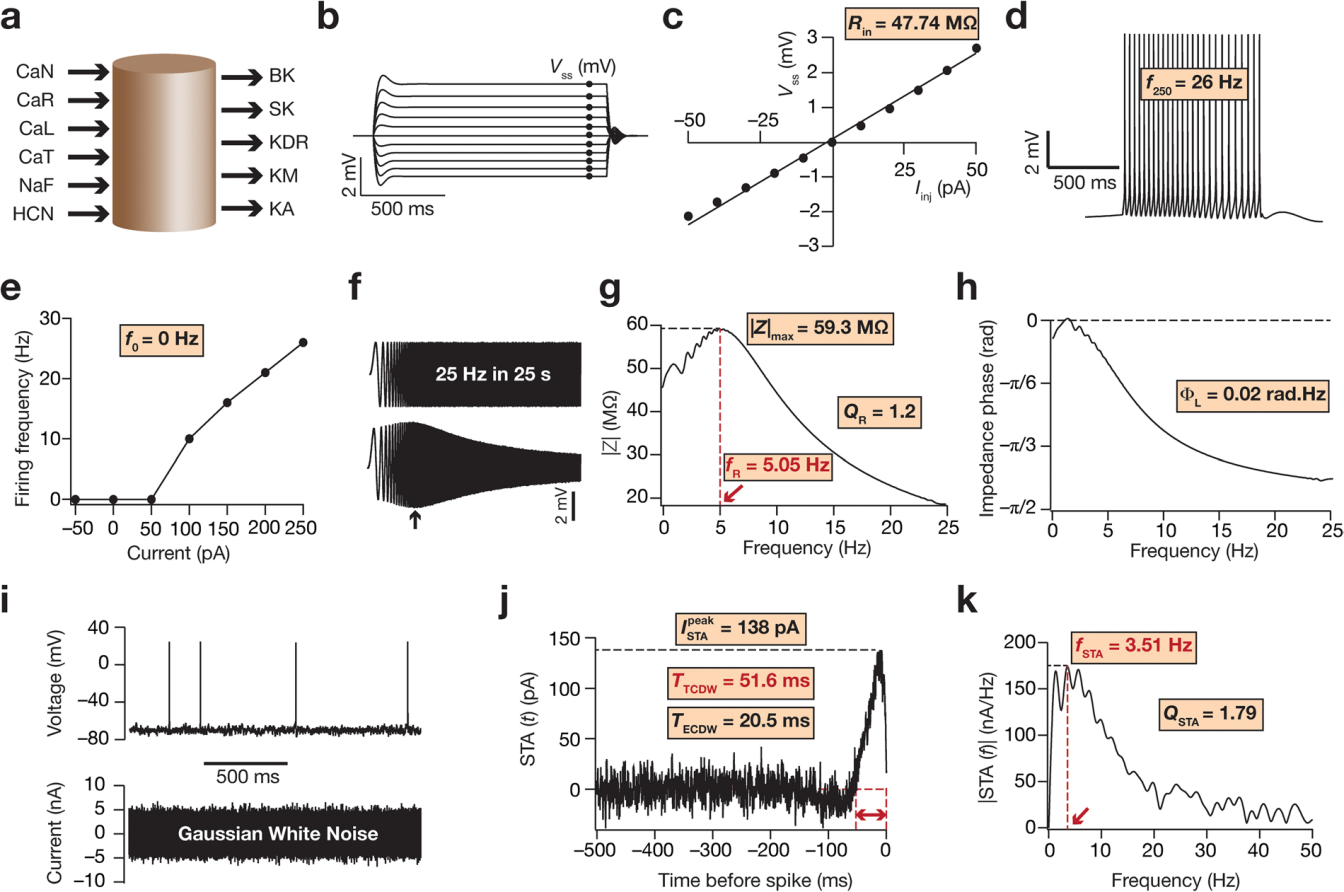
Illustration of physiological measurements employed in this study using the base model. (**a**) Depiction of the cylindrical single compartment base model, along with the ion channels that were inserted in it. Arrows pointing towards the cylinder represent inward currents and arrows pointing away from the cylinder represent outward currents. (**b**) Voltage traces in response to a 1000 ms current injection, from –50 pA to + 50 pA, in steps of 10 pA. *V*_ss_ represents the steady state voltage (black circles) achieved with each current injection. (**c**) Plot of the steady-state voltage obtained from the traces in panel **b** versus the corresponding current amplitude. Slope of the linear fit to this *V–I* curve defines input resistance (*R*_in_). (**d**) Voltage trace in response to a 250 pA current injection, lasting for 1000 ms. The number of action potentials fired during this period is measured as *f*_250_. (**e**) *f–I* curve for the base model, plotting firing frequency for different amplitudes of current injection. *f*_0_ measured the firing frequency when no current was injected. (**f**) Upper panel represents the chirp current stimulus of 20 pA amplitude (peak-to-peak), and with a frequency that increases linearly from 0 Hz to 25 Hz in 25 s. Lower panel represents the voltage response of the neuron to such a chirp stimulus. The black arrow depicts the location of maximal voltage response. (**g**) Impedance amplitude profile obtained from the voltage response to the chirp stimulus, both shown in panel **f**. |*Z*|_max_ represents the maximal impedance amplitude; resonance frequency *f*_R_ is the frequency at which |*Z*|_max_ is achieved (red arrow) and *Q*_R_ is the resonance strength. (**h**) Impedance phase profile obtained from the voltage response to the chirp stimulus, both shown in panel **f**. Total inductive area Φ_L_ is area under the positive part of this impedance phase profile. (**i**) Lower panel represents one part of the Gaussian white noise (GWN) current stimulus. Upper panel represents the voltage response of the neuron to this GWN current stimulus. (**j**) STA trace for the base model. On the *X*-axis is the time before generation of an action potential in ms, while the *Y*-axis is STA current in pA. ${I}_{STA}^{peak}$ represents the STA peak current; *T*_TCDW_ (window depicted by the red arrows) and *T*_ECDW_ represent the total and the effective coincidence detection windows, respectively. (**k**) Fourier transform of the STA from panel **j** that reveals its spectral selectivity at the STA characteristic *f*requency (*f*_STA_; red arrow). *Q*_STA_ is the selectivity strength.

A cylindrical base model was created with both length (*L*) and diameter (*d*) kept at 105 μm (Fig. 1); *R*_m_ and *C*_m_ were kept at 40 kΩ.cm^2^ and 1 μF/cm^2^ respectively, to set the passive input resistance (*R*_m_/π*dL*) to be around 120 MΩ^35^ and the passive time constant (*R*_m_*C*_m_) to be 40 ms^36^. We introduced the following ion channels, with kinetics and gating properties derived from hippocampal pyramidal neurons, into this passive structure: fast sodium (NaF), delayed rectifier potassium (KDR), *A-*type potassium (KA), *M-*type potassium (KM), hyperpolarization-activated cyclic-nuclotide gated (HCN) nonspecific cation, *L-*type calcium (CaL), *N-*type calcium (CaN), *R-*type calcium (CaR), *T-*type calcium (CaT), big- (BK) and small-conductance (SK) calcium-activated potassium channels. Conductances of the different ion channels were hand-tuned such that values for all 12 intrinsic measurements (Fig. 1b–j) fell in their appropriate experimentally determined ranges (conductance values are shown in Table 1). These base model parametric values then provided the span for the stochastic search that was performed on the different parametric combinations (Table 2). Apart from the match in excitability measurements with their respective experimental counterparts^15,35,36,38^, we noted that the STA associated with the base model (Fig. 1j) was endowed with a small negative lobe, yielding an *f*_STA_ (Fig. 1k) within the valid theta range of frequencies^25^. The base model measurements also showed that the effective coincidence detection window (ECDW) was in the gamma frequency range (Fig. 1j), another characteristic feature of the STA associated with CA1 pyramidal neuronal soma^25^.

**Table 1:** List of parameters and ranges of the uniform distributions used.

	Neuronal Parameter	Symbol	Value in base-model	Testing range
1	Specific membrane capacitance, μF/cm^2^	*C*_m_	1.00	0.75 to 1.50
2	Specific membrane resistance (*R*_m_), kΩ.cm^2^	*R*_m_	40	20 to 80
3	Decay time constant of calcium dynamics, ms	τ_Ca_	33	10 to 300
	**Maximal conductance of:**			
4	NaF channel, mS/cm^2^	*g*_NaF_	14.0	7.0 to 28.0
5	HCN channel, μS/cm^2^	*g*_HCN_	31.8	15.9 to 63.6
6	KDR channel, mS/cm^2^	*g*_KDR_	6.05	3.025 to 12.10
7	CaT channel, mS/cm^2^	*g*_CaT_	1.55	0.75 to 3.0
8	CaL channel, mS/cm^2^	*g*_CaL_	0.5	0.25 to1.0
9	CaR channel, mS/cm^2^	*g*_CaR_	0.1	0.05 to 0.2
10	CaN channel, mS/cm^2^	*g*_CaN_	0.1	0.05 to 0.2
11	KA channel, mS/cm^2^	*g*_KA_	6.06	3.03 to 12.12
12	KM channel, μS/cm^2^	*g*_KM_	0.1	0.05 to 0.2
13	SK channel, μS/cm^2^	*g*_SK_	0.01	0.005 to 0.02
14	BK channel, μS/cm^2^	*g*_BK_	0.5	0.25 to 1.0

**Table 2:** Bounds on intrinsic measurements used to define valid model population.

	Intrinsic Measurement	Lower bound	Upper bound
1	Input resistance (in MΩ)	30	90
2	Firing frequency for 0 pA current injection (in Hz)	0	0
3	Firing frequency for 250 pA current injection (in Hz)	20	35
4	Resonance frequency (in Hz)	2.0	5.5
5	Resonance strength	1.05	1.5
6	Maximum impedance amplitude (in MΩ)	50	110
7	Total inductive phase (in radian.Hz)	0	0.20
8	STA characteristic frequency (in Hz)	3.0	7.0
9	STA selectivity strength	1.5	2.2
10	Peak STA current (in pA)	90	900
11	Total coincidence detection window (in ms)	25	62
12	Effective coincidence detection window (in ms)	5	25

Electrophysiological bounds for measurements 1–7 were obtained from (Narayanan and Johnston, 2007, 2008; Narayanan *et al*., 2010 and Malik *et al*., 2016)^35,36,38,39^ and STA measurements 8–12 were obtained from (Das and Narayanan, 2017)^25^.

### Stochastic sampling and validation

We stochastically generated 5000 unique models by sampling uniform distributions for 14 parameters (all ion channel conductances, the specific membrane capacitance, and the decay time constant of calcium), with each distribution centered about the corresponding value in the base model (Table 1). Validity of each model was then ascertained by checking if *all* the 12 intrinsic measurements computed from these models fell within their respective electrophysiological ranges (Table 2). Of the 12 measurements, five were derived from the STA that characterized the operating mode and coincidence detection windows. The other measurements defined intrinsic excitability and frequency-dependent neuronal response properties in the subthreshold range. Of the 5000 models generated, we found 72 models (~1.5% of the total number of models) that satisfied all these validity criteria and were declared as valid models.

### Ion channel degeneracy in the co-emergence of STA characteristics and intrinsic excitability measurements

To address our primary question on whether degeneracy could be a substrate for neurons to both possess specific operational characteristics and maintain their intrinsic excitability properties, we assessed the parametric ranges of these 72 valid models. As a direct consequence of the validation criteria employed in our analyses, these 72 models satisfied all encoding and excitability constraints that we had placed. Were the 14 model parameters (Table 1) that defined these 72 models organized into clusters or were they distributed? A distributed set of underlying parameters, where different sets of channel conductances yield similar function, would constitute a direct line of evidence for the expression of degeneracy in this model population.

**Figure 2:**
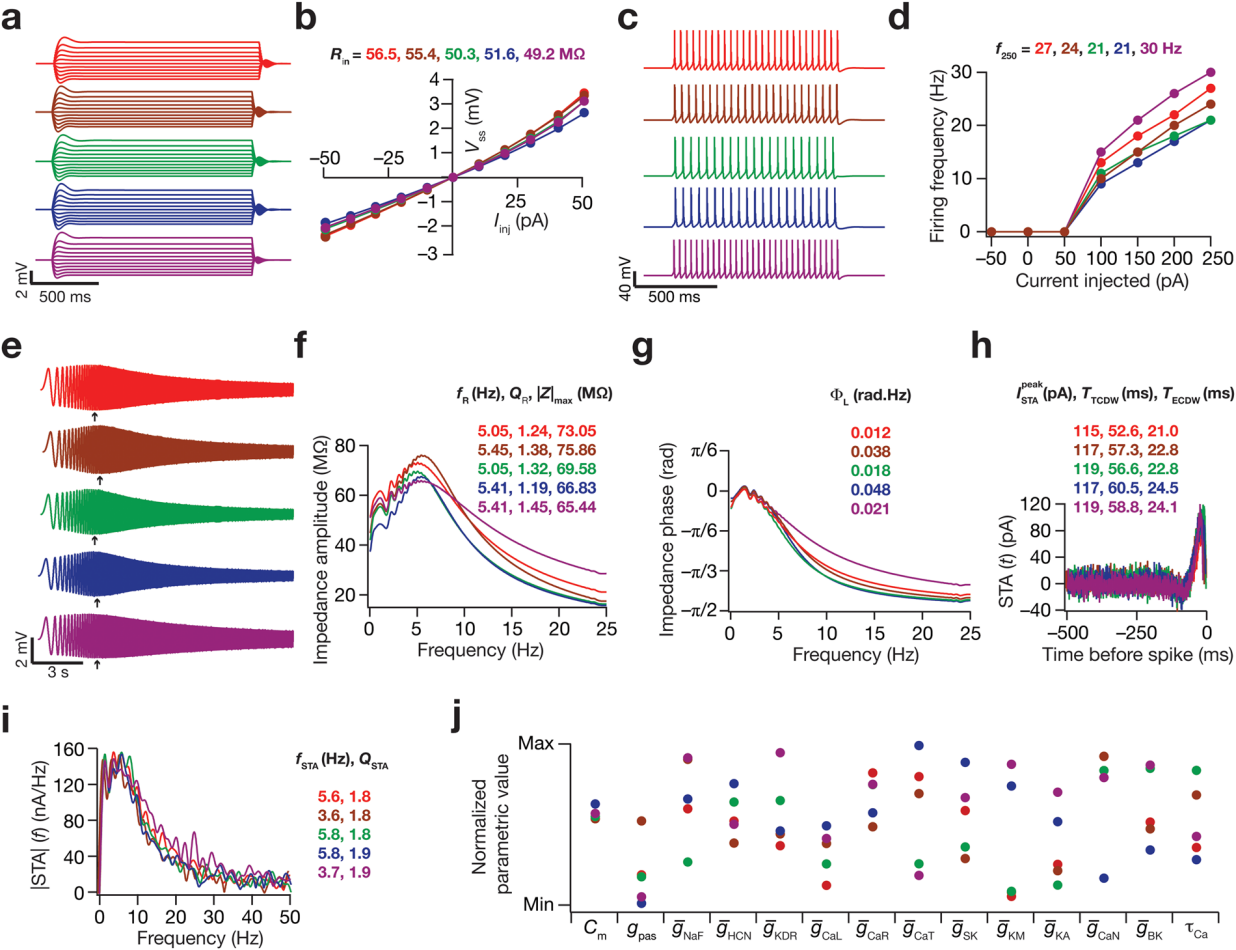
Disparate molecular-scale parameters yielded models with similar cellular-scale physiological measurements, spanning encoding and excitability characteristics. The voltage traces and parameters correspond to 5 models that satisfied all the validity criteria presented in Table 2. The color codes for model characteristics are maintained across panels. (**a**) Voltage responses of the 5 models to current injections spanning the range –50 pA to + 50 pA in increments of 10 pA. (**b**) Plot of the steady-state voltage responses obtained from panel **a** versus the corresponding current injection to obtain *V-I* curves and input resistance (*R*_in_). (**c**) Voltage responses of the 5 models to a 250 pA current injection. (**d**) The *f–I* curves for the 5 models. (**e**) Voltage traces obtained from the five models in response to the chirp stimulus. Arrows represent the locations of the maximal voltage response. (**f**) Impedance amplitude profiles for the five models, derived from the chirp stimulus responses shown in panel **e**. (**g**) Impedance phase profile for the five models, derived from the chirp stimulus responses shown in panel **c**. (**h–i**) STA traces (**h**) and their Fourier spectra (**i**) for the five models. (**j**) Distribution of the 14 parameters that governed the 5 model neurons, normalized with reference to their respective min-max ranges (Table 1).

To assess this, we chose five models whose intrinsic measurements (both excitability measurements and STA measurements) were very similar (Fig. 2a–i). We then plotted the value of each of the 14 parameters (normalized individually with reference to their respective min and max values in Table 1) for all these five models (Fig. 2j) to assess if they were clustered or were disparate across distinct models. We noted that most parametric values spanned their entire respective ranges, suggesting the absence of clustering or the need to maintain individual conductances at specific values for achieving functional similarity. This provided us with the first line of evidence that degeneracy could indeed provide a substrate for neurons to concomitantly maintain their encoding/operational characteristics along with their excitability characteristics, whereby disparate sets of channels could result in the joint emergence of both physiological signatures.

**Figure 3:**
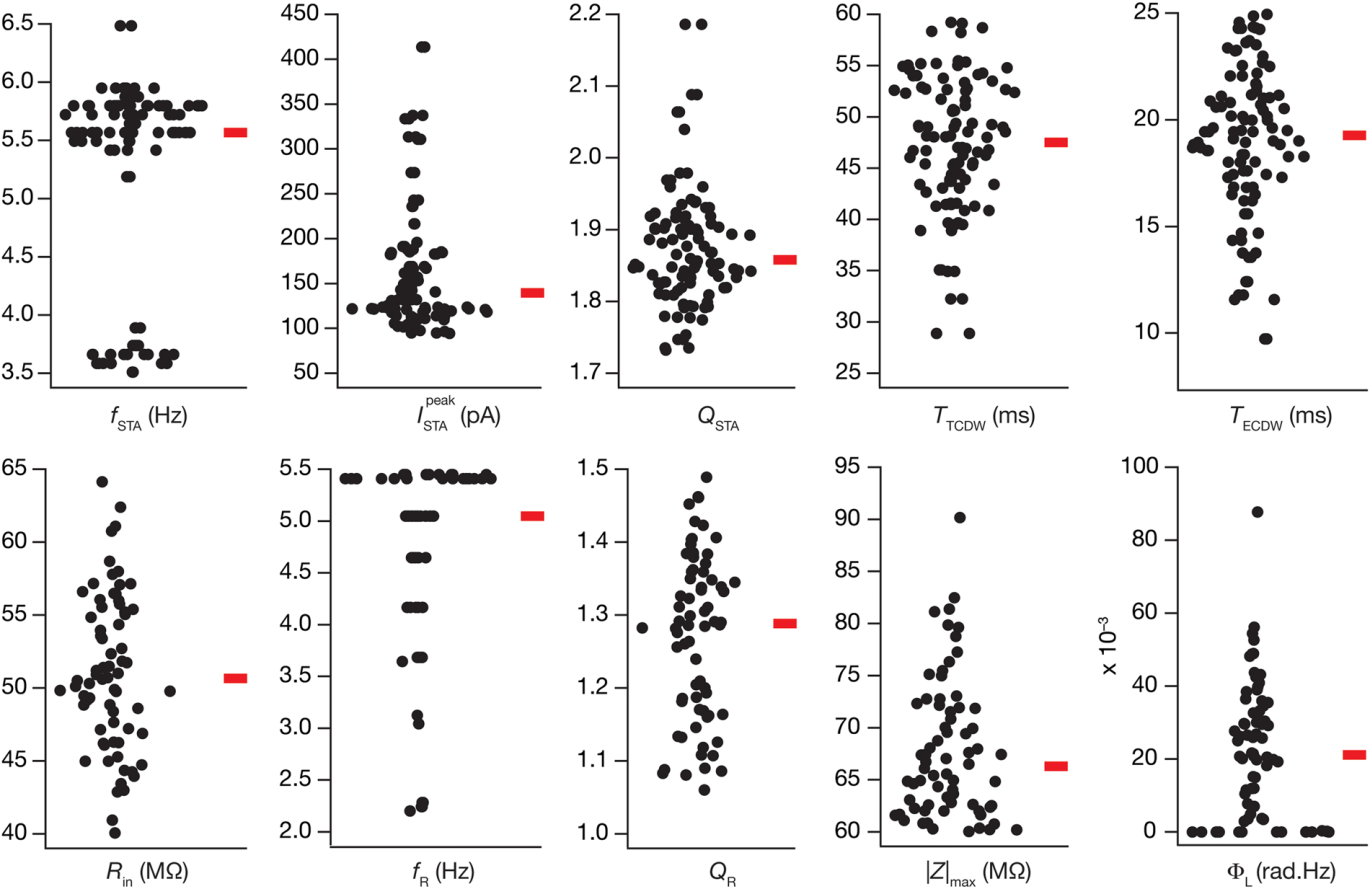
Distribution of neuronal intrinsic physiological characteristics for the valid model population. Each sub-panel depicts the population distribution of a given intrinsic measurement for the 72 valid models, shown with the median value, indicated by the red bar. Compare ranges with validity criteria specified in Table 2. The theta-frequency range of *f*_STA_ and gamma-frequency range of *T*_ECDW_ may be noted for all models.

**Figure 4:**
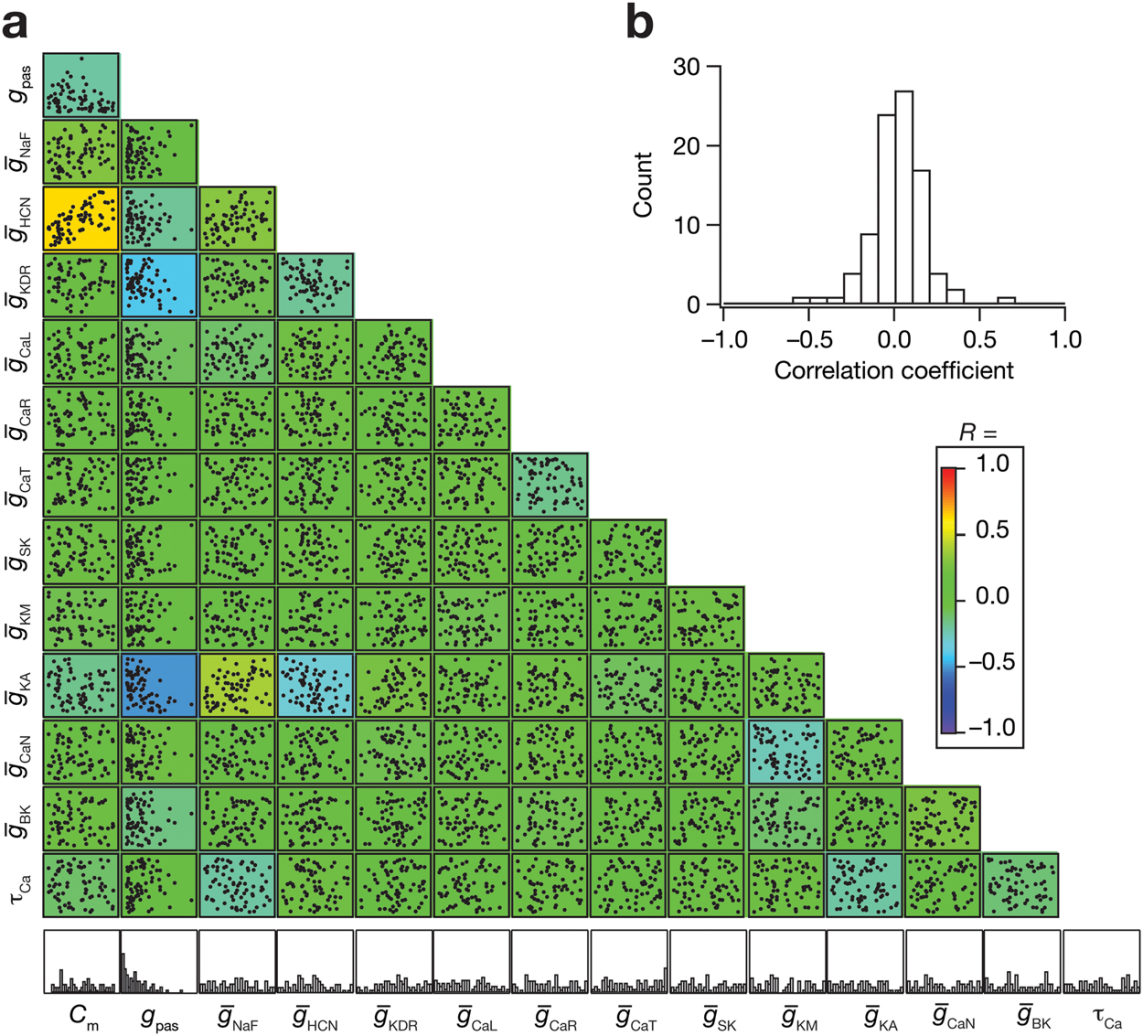
Broad distributions of and weak pairwise correlations amongst parameters of the valid model population unveiled parametric degeneracy in the concomitant expression of excitability and encoding measurements. (**a**) Lower diagonal of a matrix depicting pairwise relationships between the 14 parametric values of the 72 valid models. Each subplot is a scatter plot depicting values of the two parameters, labeled to the bottom and the left. The lowermost row comprises normalized histograms of the 14 parameters across all 72 valid models, and may be noted to have a broad distribution spanning the entire parametric range. These plots are overlaid on the lower diagonal of a color-coded matrix constructed from the respective Pearson correlation coefficient values. (**b**) Histogram of the correlation coefficients obtained from the scatter plots in panel **a**.

The choice of five hand picked similar models where there was evidence for degeneracy presents a demonstration of the expression of degeneracy with reference to these physiological measurements. Do these conclusions extend to all the valid models in the populations? To address this, we plotted the values for the 10 intrinsic measurements (excluding *f*_0_ and *f*_250_, for all the 72 valid models), and by virtue of the validation process, they fell within the appropriate experimentally determined ranges (Fig. 3), while also confirming the presence of heterogeneity in these models. However, when we plotted the histograms of individual parameters for all 72 models, we found the histograms to span the entire min-max range (Table 2) of the respective parameter (Fig. 4a, bottom most panels).

Although the widespread ranges of individual parameters strongly point to disparate parametric combinations yielding similar function, could this be because different ion channels have pairwise relationships in their conductance values? To address this, we computed pair-wise correlations across all 14 parameters for each of the 72 models (Fig. 4a–b). We found that 97% (88 out of 91) of the correlations were weak or very weak^41^, with values between –0.4 and 0.4 (Fig. 4b). These observations showed the absence of strong cross-dependencies across channel conductances and other parameters in the concomitant emergence of STA-based and excitability measurements. Together, these results demonstrate that ion channel degeneracy could be a substrate for neurons to possess specific operational characteristics and still maintain their intrinsic excitability properties.

**Figure 5:**
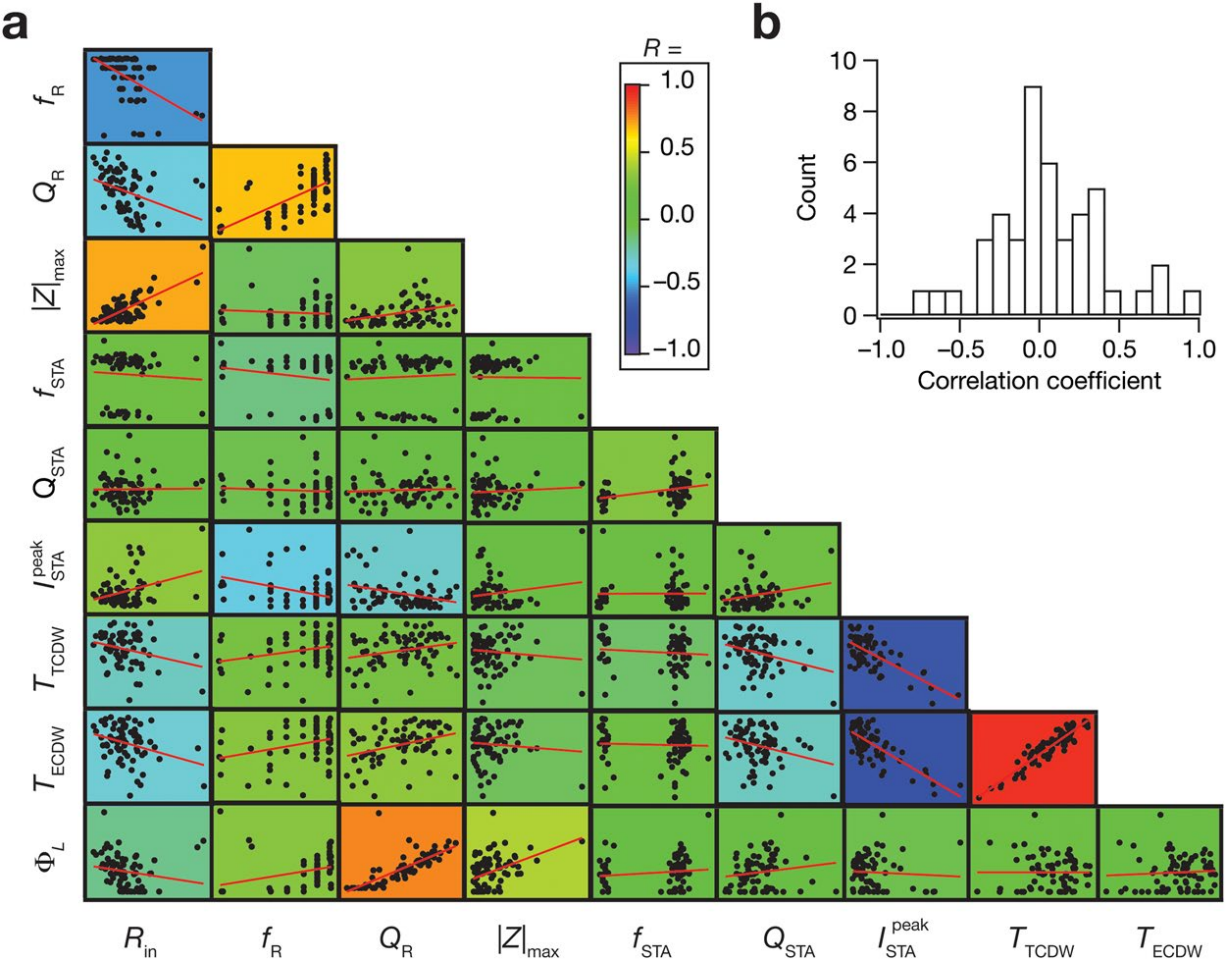
Intrinsic measurements of the valid-model population exhibited differential correlations. (**a**) Pairwise scatter plots with respective linear fits (red lines) across the 10 intrinsic measurements for the 72 valid models. These plots are overlaid on the lower diagonal of a color-coded matrix, containing the respective correlation coefficient values. (**b**) Histogram of the correlation coefficients obtained from the scatter plots in panel **a**.

Finally, we asked if there were significant cross-dependencies in the distinct measurements we had employed in our validation process. This is important because if the measurements had significant correlations between them, it would imply that we were not significantly constraining the parameters to suppress the potential expression of degeneracy. These correlations also allow us to arrive at another layer of physiological validation by comparing them with their electrophysiological counterparts^25^. To assess this, we determined the pairwise correlations amongst 10 intrinsic measurements for all the 72 valid models (Fig. 5). Consistent with previous experimental observations from CA1 pyramidal neurons^25,42^, we observed (Fig. 5) weak correlation coefficients for a large proportion of these pairwise comparisons (38 of 45, or 84%, with values between –0.4 to 0.4^41^), suggesting that there were no strong cross-dependencies across these measurements. However, in a manner consistent with prior experimental observations^25,42^, we observed strong positive correlations between excitability measurements (*R*_in_ and |*Z*|_max_) and coincidence detection metrics (*T*_TCDW_ and *T*_ECDW_). These observations demonstrate that the different measurement constraints on the model were not correlated, thereby ensuring that the model was subjected to strong constraints that reflected different functional characteristics.

If these measurements indeed reflected different functional characteristics, validity or invalidity of a specific model to one of these measurements should not be predictive of validity or invalidity of the same model to another measurement. To test if this was indeed the case, we first took the six standard intrinsic measurements (*R*_in_, *f*_250_, |*Z*|_max_, *f*_R_, *Q*_R_, Φ_L_) of all the 5000 models. For any given pair of measurements (say, A and B) from these 5000 models, we constructed three matrices assessing pair-wise dependencies of validity across these measurements: (i) the valid-valid matrix showed the number of models that were also valid for measurement A when they were valid for measurement B (Supplementary Fig. S1a); (ii) the invalid-invalid matrix depicted the number of models that were also invalid for measurement A when they were invalid for measurement B (Supplementary Fig. S1b); and (iii) the valid-invalid matrix provided number of models that were valid for measurement A, but were invalid for measurement B (Supplementary Fig. S1c). If these measurements were redundant in terms of their selection pressure on the model validation process, then validation/invalidation for one measurement should predict the validation/invalidation of the other redundant measurement. However, in our case, we found from these matrices that validity (or invalidity) for one measurement did not predict validity (or invalidity) for any of the other intrinsic measurements.

We repeated these analyses with the 5 STA measurements for the 355 (of the total 5000) models that satisfied all the intrinsically physiological criteria (Supplementary Fig. S1d–f), and arrived at similar conclusions for STA measurements as well. In addition, these analyses also demonstrated that there were models that were valid for all excitability measurements, but did not satisfy validity constraint for one or the other STA measurement (Supplementary Fig. S1d,f). Together, our analyses demonstrated that the set of measurements employed in this study were not redundant in terms of their selection pressure on the model validation process.

### Virtual knockout models unveiled a many-to-many relationship between channels and STA measurements

**Figure 6:**
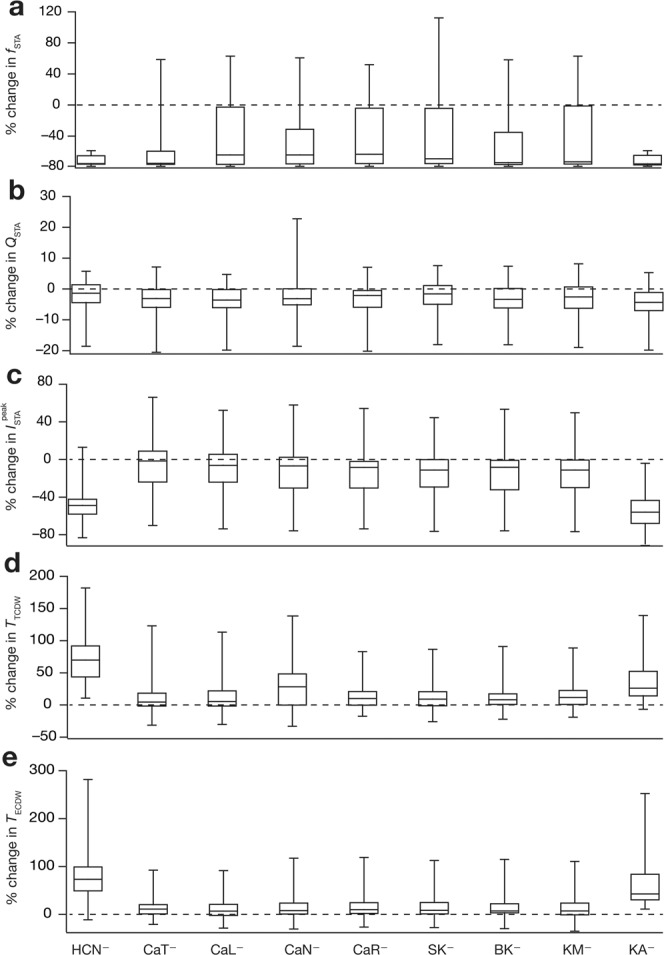
Virtual knockout simulations unveiled a many-to-many relationship between channel subtypes and intrinsic measurements. Distributions of percentage changes (represented as quartiles) for STA characteristics frequency *f*_STA_ (**a**), STA selectivity strength *Q*_STA_ (**b**), peak STA current ${I}_{{\rm{STA}}}^{{\rm{peak}}}$ (**c**), total coincidence detection window *T*_TCDW_ (**d**) and effective coincidence detection window *T*_ECDW_ (**e**). Each column corresponds to a specific channel knockout, as indicated by the label at the base of the figure. Also see Supplementary Figs. S2–S5.

Virtual knockout models (VKM) are an ideal way to assess the impact of individual channels on physiological measurements within the degeneracy framework that involve heterogeneous populations^9,14,40,43^. For each valid model, we generated virtual knockouts (by setting the respective conductance to zero, without altering any other parameter) with reference to 9 ion channels (the spike-generating conductances were not knocked out, given that they are essential for STA computations): CaT, CaR, CaN, CaL, KM, KA, SK, BK and HCN (Supplementary Fig. S2). We compared the 5 STA-based measurements of every VKM (across 9 channels) with the corresponding baseline values (Supplementary Figs. S3–S5). For all VKMs (corresponding to each of the 9 channels), we found all STA measurements to be significantly different (Wilcoxon rank sum test, *p* < 0.05) compared to the corresponding values in their respective base models (Supplementary Figs. S3–S5). From these analyses, we noted the following (Fig. 6, Supplementary Figs. S2–S5): (i) the impact of knocking out different channels on specific measurements was differential, whereby different channels had distinct effects on these measurements; (ii) for a given ion channel and a given measurement, there was significant variability in the impact of the knockout, even involving the direction of change in some cases; and (iii) knocking out a specific channel altered several STA measurements and any given measurement was altered with knockout of several channels, implying a many-to-many relationship between channel conductances and STA measurements.

To assess the specifics of the impact of channels on STA measurements, we plotted the distribution of the knockout-induced changes in each STA measurement for each of the 9 different channels (Fig. 6). Although there was significant heterogeneity in the impact of different channels, we noted that the HCN and KA VKMs resulted in a reduction, relative to their respective pre-knockout values, of >60% in the *f*_STA_ across models (Fig. 6a). We noted this to be consistent with previous computational and electrophysiological studies on hippocampal pyramidal neurons, which have demonstrated that blocking HCN channels reduces its *f*_STA_^23–25^. We also noted that no particular ion channel caused either a consistent reduction or augmentation of the *Q*_STA_ (Fig. 6b).

We observed that the peak STA current (${I}_{{\rm{STA}}}^{{\rm{peak}}}$) was consistently lower in the case of the HCN and KA VKMs, with VKMs of other channels showing considerable variability with reference to their impact on ${I}_{{\rm{STA}}}^{{\rm{peak}}}$ (Fig. 6c). As ${I}_{{\rm{STA}}}^{{\rm{peak}}}$ is a measure of excitability with lower ${I}_{{\rm{STA}}}^{{\rm{peak}}}$ (*i.e*. the average current required for triggering an action potential is lower) translating to higher excitability, these observations imply that the presence and activation of either channel reduces the neuron’s excitability^23–25^. However, around half of the CaT VKMs showed an increase in the peak STA current compared to their baseline values, with the other half manifesting a decrease after knockout of CaT channels. We noted that this could be an effect of the expression of SK channels in our model. In models where the SK channel density was larger, the knockout of CaT channels would lead to a significant reduction of SK current, thereby leading to an increase in excitability, whereas in models where the SK channel density was lower, the knockout of CaT channels would express directly as a reduction in a depolarizing current, which manifests as a reduction in excitability. A similar explanation holds for the knockout of other calcium channels as well (Fig. 6c).

Finally, with respect to both measures of coincidence detection (Fig. 6d–e), *T*_TCDW_ and the *T*_ECDW_, the HCN and KA VKMs showed a large, consistent increase with respect to the corresponding baseline values. In the case of the HCN VKMs, these findings tally with prior single-sensitivity computational results from hippocampal pyramidal neurons^23,24^. The other VKMs exhibited small increases in *T*_TCDW_ and *T*_ECDW_.

## Discussion

Our study has demonstrated (through biophysically and physiologically constrained models) that neurons possessing (1) similar encoding characteristics (in terms of the specific set of measurements from the STA kernels), (2) similar operating characteristics (in terms of whether they are coincidence detectors or integrators) and (3) similar excitability characteristics (both frequency-dependent and independent) can exist in the absence of individual or pair-wise channel homeostasis. These results suggest that hippocampal neurons have significant degrees of freedom to achieve the specific encoding and homeostasis targets associated with their physiology; in other words, degeneracy (at the molecular level) can enable cells to exhibit similar encoding and homeostasis (functional properties at the cellular level).

### Implications for degeneracy in the emergence of spike-triggered average

Our study provides lines of evidence for the *existence* of different structural routes, involving different sets of ion channels, to achieve similar spike-triggered average and similar excitability characteristics. The many-to-many mapping between ion channels and functional measurements (Fig. 6) provide the substrate for the existence of these different solutions. The stochastic search strategy that we employed to arrive at this evidence for expression of degeneracy, however, does not address the question of how neurons organize their channel densities to arrive at these distinct solutions. It has been postulated that a general requirement for systems manifesting degeneracy is a closed-loop continual *error-correction feedback signal* that conveys whether a specific functional outcome is being achieved or not. This error signal is then employed by the system to organize its parametric space to achieve a given function, with the specific structural components chosen to achieve the function determined by the internal state of the system, neuromodulatory tones and the nature of afferent signals. A specific instance of such a broad description is with reference to the role of calcium homeostasis in maintaining channel densities through calcium-dependent transcription that is dependent on a calcium error signal^7,8,10,15,44^. Here, it has been shown that the solutions achieved by neurons and their networks could recruit disparate channels and receptors depending on the specific components of the network and the nature of afferent signals^15,44^. Although calcium constitutes one possible error correcting feedback mechanism, other possibilities including membrane voltage^45^ and firing rate^46^ could act as conduits for conveying such error signals involving their homeostasis^47,48^. The specific closed-loop feedback pathway could involve transcriptional networks, neuronal networks regulating excitation-inhibition balance and well-established interaction motifs widely expressed in these network structures^49–51^.

A neuron in particular, or a biological system in general, executes different functions in different contextual settings, with the context defined by the state of the system and the nature of external inputs. Physiological measurements, including those of excitability or operating mode, are designed to assess specific characteristics of neurons. It has been shown in several scenarios that the ability of a neuron to maintain one measurement does not necessarily translate to its ability to maintain another. For instance, there are scenarios where calcium homeostasis is maintained without homeostasis of firing rate or firing pattern, or scenarios where one model measurement is observed to match electrophysiological counterparts but others don’t^15,44^. This *dissociation* in different modes of homeostasis implies that models have to explicitly account for different measurements, and tuning of one of them doesn’t necessarily maintain all measurements at physiological levels^8,15^. Such dissociation also implies that multi-parametric stochastic searches would, in general, yield lesser *valid models* as additional physiological objectives are added to the validation process (e.g., Supplementary Fig. S1).

In our model, for instance, we employed 12 intrinsic measurements (Table 2) to validate models sampled through stochastic search. In this process, owing to the dissociation of different forms of homeostasis, there were models that satisfy 11 of the measurements, but not the 12^th^ measurement. Does this imply that the degeneracy reported here would reduce as additional physiological constraints are included to the validation process? Degeneracy refers to the ability of different structural components to elicit similar function, and is by definition specific to the function in hand^1,8^. Degeneracy also implies that the same structure could be involved in the execution of a *different function* under a different context. Based on these definitions, it is evident that the presence of additional functional constraints on the models certainly reduces the number of valid models that satisfy all the constraints; however, this doesn’t necessarily translate to reduction or abolition of degeneracy with reference to specific functions or their combinations.

The expression of degeneracy is dependent on the ability of distinct structural components to alter a specific function, implying that the presence of a many-to-one relationship between structures and a specific function is a *sufficient substrate* for the expression of degeneracy. In our case, we observe that several channels alter each functional measurement assessed (Fig. 6), implying the expression of degeneracy for each of these *individual functional measurements*. In addition, we also note that changes in each of the individual channels alter several functions in a manner that is dependent on the model parameters, suggesting a many-to-many relationship between structure and function. Furthermore, in our analyses, we have employed only a subset of parameters available for the neuron in regulating its functions. If the stochastic search space were to be increased to account for these additional parameters (other ion channels, receptors, neuromodulators, afferent input patterns, etc.), and heterogeneities therein, then the degrees of freedom available to the neuron to achieve *specific functional measurements* also increase in combinatorial fashion (owing to strong interactions between constituent components). This explosion in degrees of freedom then provides additional routes to fulfilling the multiple functional constraints (either in the same context or under different contexts). Finally, the cell-to-cell and animal-to-animal variability in constituent components, despite the expression of similar functional outcomes, provide lines of evidence for the expression of degeneracy in biological systems^1,7,8,16,17,52^.

### Impact of individual ion channels on spike-triggered average

Our analyses show that removal of any of the 9 ion channels in any of our valid models causes the model to shift its operating mode to the integrator end of the integrator-coincidence detection continuum. This would indicate that interactions amongst the ion channels modulate the STA-based properties of a neuron. However, the HCN and KA ion channels were found to be the dominant regulators of a CA1 neuron model’s operation mode, since absence of either ion channel imparts pronounced integrator-like traits, such as very low *f*_STA_ (~1 Hz) and broad coincidence detection windows.

The HCN channel has been shown to impart coincidence detector traits to hippocampal CA1 neurons^23,24^. Our results are consistent with these studies, as HCN VKMs lacked coincidence detector traits. On the other hand, an increase in KA conductance has been shown to cause an increase in the resonance frequency^42^, but results in a decrease in the *f*_STA_^24^. Our observations, however, suggest that eliminating the KA conductance results in a consistent reduction in *f*_STA,_ while also resulting in a broadening of the coincidence detection windows. The dependence of frequency-dependent and frequency-independent excitability measurements on different ion channels^13–15,42^, the role of different ion channels in regulating STA measurements^23–25^, and the dissociation between subthreshold resonance and STA spectral selectivity^24–26^ in hippocampal pyramidal neurons have been analyzed earlier. Although an earlier study^24^ predicts an increase in *f*_STA_ with a reduction in KA channel density, the model employed there did not contain all the different ion channels expressed by hippocampal neurons and therefore do not account for the several cross-channel interactions that manifest in our analyses. In addition, as the excitability of the neuron increases with the elimination of KA channels^42,53^, a lower variance was required for the Gaussian white noise to elicit an average firing frequency of 1–1.5 Hz. As *f*_STA_ is known to be directly related to this variance^23–25^, the observed reduction in *f*_STA_ in our analyses could also be explained by the reduction of the variance of the Gaussian white noise.

As CaT channels also mediate resonating conductances^54^, the CaT VKMs would have been expected to respond in a manner similar to the HCN VKMs^24^. However, our simulations indicate that the HCN conductance plays a more dominant role than the CaT conductance in mediating a CA1 pyramidal neuron’s operating mode. This could partly be attributed to the absence of calcium-activated potassium channels in the earlier study^24^. Calcium-activated potassium channels critically interact with calcium channels in regulating excitability of hippocampal pyramidal neurons^55^, and therefore could suppress the impact of CaT channels in significantly altering neuronal operational modes^56^. These postulates – about the role of cross-interactions among channels in regulating neuronal STA and the impact of the variance of Gaussian white noise in the assessment of the impact of KA channels on STA properties – could be tested with electrophysiological experiments coupled with appropriate pharmacological blockers.

Although our analyses systematically showed the dependence of specific STA measurements on individual ion channels by eliminating individual channels (Fig. 6), there were no rules or mechanisms to account for such ion channel perturbations towards compensation of specific functions. In other words, our analyses were on the acute impact of ion channel removal, with no rules to govern potential changes under chronic knockout scenarios^44^. Future studies could incorporate specific rules involving closed-loop feedback mechanisms correcting functional errors (see above) to assess the role of different channels in compensating for specific knockouts. Such analyses would reveal the dynamical impact of ion channel knockouts, and potential heterogeneities in such impact consequent to baseline heterogeneities in ion channel conductances in specific models^44^.

In summary, in this study, we demonstrated the expression of degeneracy in the concomitant emergence of excitability as well as encoding measurements in hippocampal pyramidal neurons. This demonstration was constrained biophysically by the ion channel gating and kinetic properties and physiologically validated by STA and excitability measurements, all of which were obtained from hippocampal pyramidal neurons. Our results suggest that hippocampal neurons have significant degrees of freedom to achieve the specific encoding and homeostasis targets without cross-interferences in these processes^8^. Although our conclusions could potentially extend to other neuronal subtypes, such extrapolations should specifically account for biophysical characteristics of ion channels expressed in those neurons and the STA and excitability measurements from there as well. Future studies could assess the potential of degeneracy in concomitantly maintaining encoding as well as excitability characteristics, especially with reference to physiological and pathological perturbations.

## Methods

### Neuronal base model and ion channels

A single compartmental cylindrical base model was created with length (*L*) and diameter (*d*) both equal to 105 μm. The specific membrane resistance (*R*_m_) and specific membrane capacitance (*C*_m_) — that constitute passive properties of the model — had values of 40 kΩ.cm^2^ and 1 μF/cm^2^, respectively. Twelve different types of ion channels (including leak channels with conductance *g*_leak_ = 1/*R*_m_) were inserted into this structure, with default values of channel conductances listed in Table 1. The channels employed in this study are: fast sodium (NaF), delayed rectifier potassium (KDR), *A-*type potassium (KA), *M-*type potassium (KM), hyperpolarization-activated cyclic-nuclotide gated (HCN) nonspecific cation, *L-*type calcium (CaL), *N-*type calcium (CaN), *R-*type calcium (CaR), *T-*type calcium (CaT), big- (BK) and small-conductance (SK) calcium-activated potassium channels. The reversal potentials for K^+^ and Na^+^ were kept at –90 mV and + 55 mV, respectively, and the reversal potential for the HCN conductance was set to –30 mV. The kinetics for different channels were constrained by respective electrophysiological measurements in hippocampal neurons for each of the channels present in our model: NaF, KDR and KA channels^27,28,30^, HCN channels^29^, CaR and CaL channels^27,57^, CaT channels^33^, KM channels^33,58^, BK channels^59^, and CaN and SK channels^60^. The evolution of intracellular calcium as a function of calcium current through the voltage-gated calcium channels and its buffering was modeled as^61–63^:

$$\frac{d{[Ca]}_{i}}{dt}=-\frac{10000\,{I}_{Ca}}{36\,dpt\,F}+\frac{{[Ca]}_{\infty }-{[Ca]}_{i}}{{\tau }_{Ca}}$$

where *F* is Faraday’s constant, the default value of the calcium decay time constant ${\tau }_{Ca}$ = 33 ms (Table 1), *dpt* = 0.1 µm is the depth of the shell, and [*Ca*]_*∞*_ = 100 nM is the steady state value of [*Ca*]_*i*_.

### Intrinsic properties

We employed 12 intrinsic physiological measurements to define and validate models (Fig. 1). Of these, 7 intrinsic measurements were excitability and frequency-selectivity measures that have been used to characterize hippocampal CA1 pyramidal neurons^13–15,29,35,36,38,39,42^. The other 5 measurements were STA-derived measurements, and were taken from their prior definitions^23–25^.

The 7 standard intrinsic measurements calculated were the input resistance (*R*_in_, in MΩ), firing frequency for a pulse-current injection of amplitude 0 pA and 250 pA (*f*_0_ and *f*_250_ respectively; both in Hz), the maximum impedance amplitude (|*Z*|_max_, in MΩ), resonance frequency (*f*_R_, in Hz), the resonance strength (*Q*_R_, dimensionless) and total inductive phase (Φ_L_, in radian.Hz). *R*_in_ was measured by noting the steady-state value of the membrane potential (*V*_ss_) in response to sustained current input of 500 ms duration; the injected current (*I*_inj_) was varied from +50 pA to –50 pA in steps of 10 pA. The slope of a linear fit to the plot of *V*_ss_ *vs. I*_inj_ was taken to be the input resistance. Firing frequency was assessed by measuring the number of action potentials fired in a 1000 ms interval when a sustained pulse current was injected. In particular, firing frequency was noted for injected current values set to 0 pA (*f*_0_) and when it was 250 pA (*f*_250_). *f*_0_ was measured to ensure that the model was not spontaneously spiking, an electrophysiological signature of CA1 pyramidal neurons^35,38^. To measure impedance related properties, a chirp stimulus (of constant 20-pA amplitude, but whose frequency increased linearly from 0 Hz to 25 Hz over a 25 s interval), was injected into the model^34,35^. The voltage response of the neuron model was recorded, and its Fourier transform was taken, along with the Fourier transform of the injected chirp current input. The Fourier transform of the voltage response was divided by that of the injected current to give the impedance. The magnitude of this impedance *Z*(*f*) was computed as:

1 $$|Z(f)|=\sqrt{{(Re(Z(f)))}^{2}+{(Im(Z(f)))}^{2}}$$

As *Z*(*f*) is a complex-valued function, *Re*(*Z*(*f*)) represents the real part of this function, while *Im*(*Z*(*f*)) constitutes the imaginary part. The maximum value of |*Z*(*f*)| was defined as |*Z*|_max_. The frequency at which |*Z*(*f*)| reached |*Z*|_max_ was called *f*_R_, the resonance frequency. The quality factor or resonance strength, *Q*_R_, was calculated to be the ratio of the maximum impedance amplitude and the impedance amplitude at 0.5 Hz^34,35^. The impedance phase was calculated as:

2 $$\phi (f)={\tan }^{-1}\frac{Im(Z(f))}{Re(Z(f))}$$

The area under the inductive (positive) portion of the curve is termed the total inductive phase, and was computed as^36^:

3 $${\Phi }_{L}={\int }_{\phi (f) > 0}\phi (f)df$$

The STA-derived measurements were computed from prior definitions^23–25^: the STA characteristic frequency (*f*_STA_, in Hz), the quality of frequency selectivity in the STA (*Q*_STA_), the peak STA current (${I}_{{\rm{STA}}}^{{\rm{peak}}}$, in pA), the total coincidence detection window (*T*_TCDW_, in ms) and the effective coincidence detection window (*T*_ECDW_, in ms). To determine these measurements, we first calculated the STA of each model. We did this by injecting Gaussian white noise current (Fig. 1i), whose standard deviation was adjusted to obtain a neuronal firing rate of 1–1.5 Hz^21,23–25,64,65^. We harvested current input from spike onset till 500 ms prior to it. These segments of the current input were averaged for at least 800 spikes to yield the STA. ${I}_{{\rm{STA}}}^{{\rm{peak}}}$ was defined as the peak STA current in the temporal representation of the STA. We took the Fourier transform of the STA, and found the characteristic frequency (*f*_STA_) corresponding to the peak of the Fourier magnitude. The strength of selectivity *Q*_STA_ at this characteristic frequency was calculated as the ratio between the peak STA magnitude |STA (*f*_STA_)| and |STA (0.5 Hz)|. The *T*_TCDW_ was calculated as the temporal distance between the action potential (*i.e*., *t* = 0 on the temporal representation of the STA) and the point at which the STA curve first crossed zero from the action potential. The *T*_ECDW_ was measured by weighting the *T*_TCDW_ by the amplitude of the STA within the positive region proximal to the action potential, and was computed as^23–25^:

4 $${T}_{{\rm{ECDW}}}=\sqrt{\frac{{\int }_{-{T}_{{\rm{TCDW}}}}^{0}{t}^{2}{{\rm{STA}}}^{2}(t)dt}{{\int }_{-{T}_{{\rm{TCDW}}}}^{0}{{\rm{STA}}}^{2}(t)dt}}$$

Action potential amplitude (*V*_AP_, in mV) was measured as the difference between the resting membrane potential and that at the peak of an action potential.

### Multi-parametric, multi-objective stochastic search (MPMOSS)

Several search strategies have been employed to assess and reveal the manifestation of degeneracy in the emergence of cellular scale physiological properties from molecular scale components^4,6–9,11–18,40,43,66,67^. Of these, the multi-parametric, multi-objective stochastic search (MPMOSS) technique involves random sampling to study how different combinations of molecular-scale parameters (channel conductances, channel kinetics, calcium buffering properties, etc.) derived from specific neurons could result in the emergence of signature intrinsic measurements of these neurons. If disparate sets of multiple molecular-scale parameters (for a population of neuron models) result in the robust manifestation of multiple physiological measurements (objectives) that reflect similar neuronal physiology, this model population is degenerate with respect to the measurements^8^. On the other hand, specific clusters of underlying parameters with strong correlations across parametric values would point to a strongly constrained system that lacks degeneracy. It is important, however, to note that in the latter scenario, declaring the absence of degeneracy is difficult because the stochastic search might not have spanned all stretches of the multi-dimensional parametric space.

The diverse set of models with randomized parameters thus generated was then subjected to validation. To do this, we sampled 14 parameters (all 12 ion channel conductances, the specific membrane capacitance and the decay time constant of calcium dynamics) from independent uniform distributions spanning a range that was typically 0.5× –2.0× of their respective base model values (Table 1). All other parameters, including the dimensions of the neuronal model, the reversal potentials and channel kinetics, were kept fixed across all randomized models. We generated 5000 unique models employing such stochastic sampling, with each of the 14 parameters randomly picked from the respective distributions. We computed all the 12 intrinsic measurements from each of these 5000 models. Finally, we determined the validity of each model by checking if all the 12 intrinsic measurements of the model fell within corresponding experimentally obtained bounds for hippocampal CA1 pyramidal neurons (Table 2). Apart from these 12 validation criteria, we imposed an additional constraint, in that the action potential amplitude *V*_AP_ had to be >95 mV for the model to be valid.

### Virtual knockout model (VKM) analysis

For each model, we created a virtual knockout (for a specific ion channel) by setting the conductance of that ion channel to zero. We then determined this VKM’s STA, using the techniques outlined above and computed the same five STA-based measurements – *f*_STA_, *Q*_STA_, ${I}_{{\rm{STA}}}^{{\rm{peak}}}$,*T*_TCDW_ and *T*_ECDW_. We compared these VKM-derived measurements with the corresponding measurements from the same model before the knockout. Statistics from comparative analyses involving all 72 models were then used to glean insights about the role of specific ion channels in specific STA measurements. It should be noted that these analyses assess the impact of *acute knockouts*, and do not incorporate rules for compensations after channel knockouts^44^.

Each of the neuron models we used for these analyses possessed 11 active ion channels (excluding the leak conductance); however, VKMs of both the NaF and the KDR ion channels were not generated. This is because knocking out either NaF or KDR severely impaired a neuron’s ability to fire action potentials, as these ion channels play critical roles in the depolarization and repolarization phases respectively of a spike. Thus, in the absence of spikes, computation of STA becomes infeasible. Consequently, VKM analyses were carried out for each of the other nine active ion channels. We thus generated a total of 9 × 72 = 648 VKMs. Of these, 13 VKMs could not maintain a 1–1.5 Hz firing rate for at least a 800 s interval (as these models entered into depolarization-induced block), while another subset of models (comprising 14 VKMs) generated supra-threshold oscillations even with the injection of extremely low-variance Gaussian white noise. Both these groups of models were excluded from our analyses.

### Simulation details

All simulations were performed in the NEURON simulation environment^68^ with the resting membrane potential set at –65 mV, and the temperature kept at 34 °C. All simulations were run with a step size of 25 μs. Data analysis was carried out using Igor Pro (WaveMetrics, Inc) and statistical analyses were performed using the R statistical package^69^.

## Supplementary information

Supplementary Figures S1-S5.

## Data Availability

All data generated or analyzed during this study are included in this published article and its Supplementary Information files. The codes employed in this study are available from the corresponding author on request.
