# Supplementary material for: Degeneracy in the emergence of spike-triggered average of hippocampal pyramidal neurons
Source: Sci Rep. 2020 Jan 15;10:374. doi: 10.1038/s41598-019-57243-8 (PMC6962224; doi:10.1038/s41598-019-57243-8)
Supplement: Supplementary file 1 — Supplementary Figures S1-S5. [file 41598_2019_57243_MOESM1_ESM.pdf]

# Degeneracy in the emergence of spike-triggered average of hippocampal pyramidal neurons

**Abha Jain and Rishikesh Narayanan**

*Cellular Neurophysiology Laboratory, Molecular Biophysics Unit,*

*Indian Institute of Science, Bangalore, India.*

## SUPPLEMENTARY INFORMATION

Supplementary Figure S1 ........................................................ 2

Supplementary Figure S2 ........................................................ 3

Supplementary Figure S3 ........................................................ 4

Supplementary Figure S4 ........................................................ 5

Supplementary Figure S5 ........................................................ 6

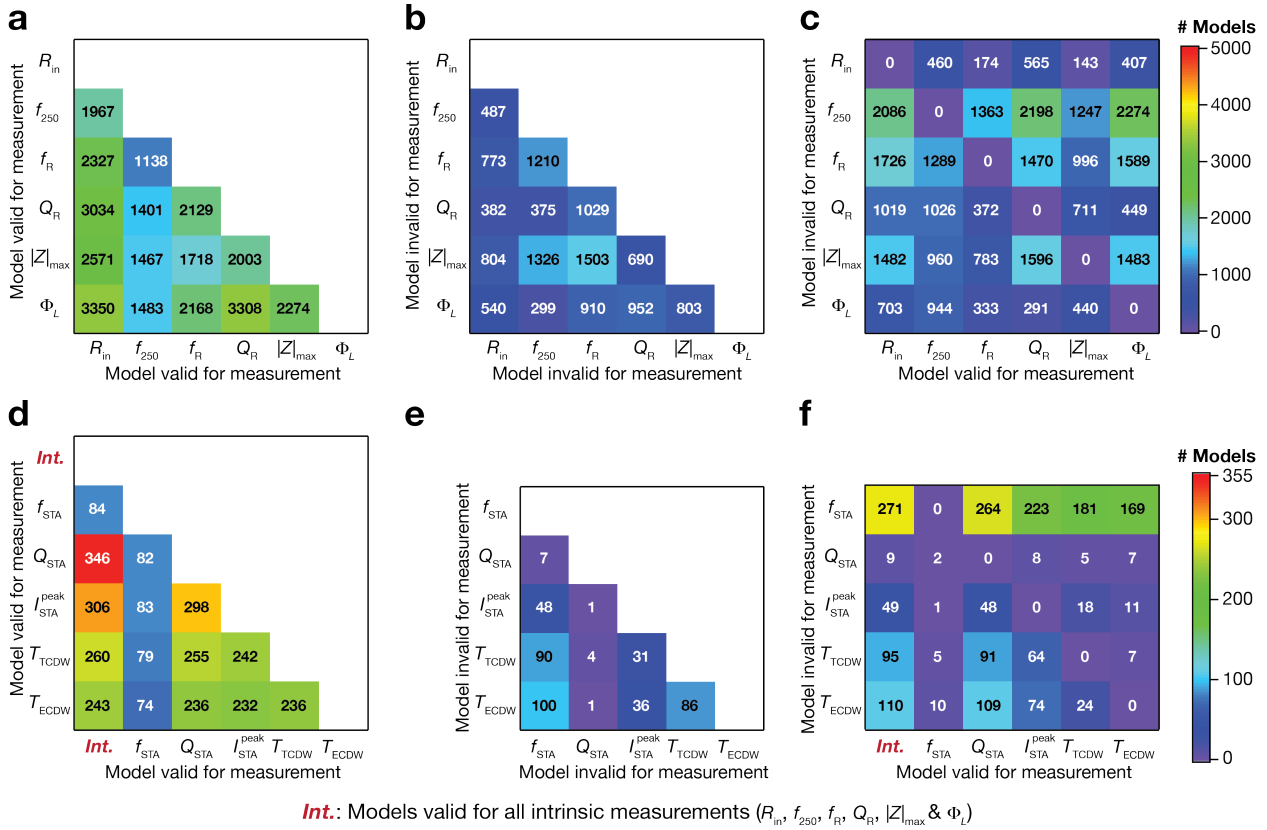


**Supplementary Figure S1:** Assessment of pair-wise dependencies of validity for measurements employed in this study. **(a)** Matrix representing pairwise count of models that were simultaneously valid for two of the 6 intrinsic physiological measurements ($R_{in}$, $f_{250}$, $|Z|_{max}$, $f_R$, $Q_R$, $\Phi_L$). **(b)** Matrix representing pairwise count of models that are simultaneously invalid for two of the 6 intrinsic physiological measurements measurements. **(c)** Matrix representing pairwise count of models that are simultaneously valid for one intrinsic measurement and invalid for another of intrinsic measurements. **(a–c)** were performed for all the 5000 models. $f_0$, the spontaneous firing rate was identically zero for all 5000 models, and therefore is not depicted here. **(d–f)** Same as panels (a–c), but for the 355 models that satisfied validation criteria for the 7 intrinsic physiological measurements (represented as *Int*). The validation criteria for all the intrinsic and STA measurements are provided in Table 2.

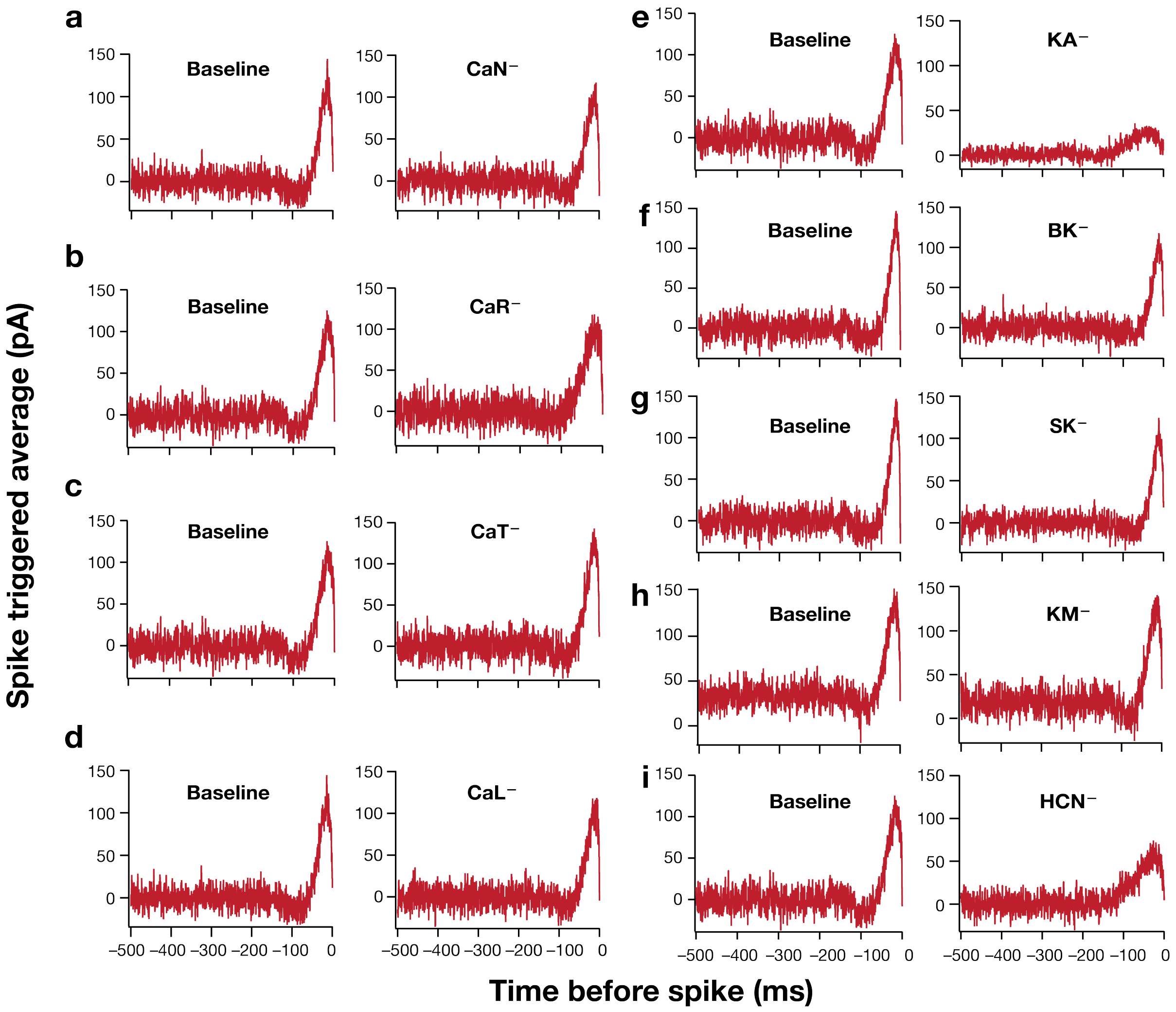


**Supplementary Figure S2:** A qualitative depiction of how knocking out specific ion channels affects a neuron's spike triggered average (STA). Each subpanel compares a neuron's STA under baseline conditions (left) with that of the same model lacking a specific ion channel (right); the ion channel that has been knocked out is indicated in each subpanel. For every graph, the *X*-axis is time before the generation of an action potential (in ms), while the *Y*-axis is STA current (in pA).

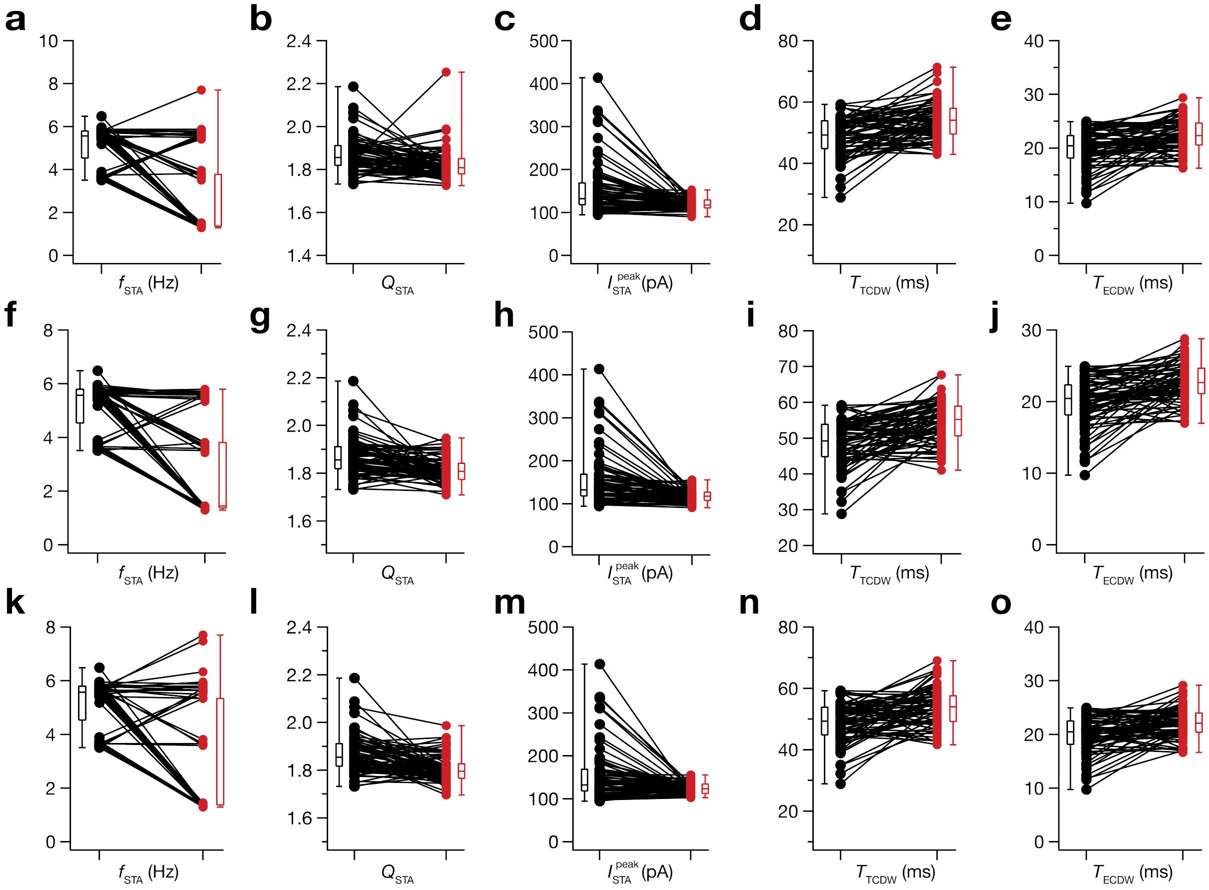


**Supplementary Figure S3:** Comparison of the five STA-based properties between the baseline models and the VKMs. In each subpanel, the black dots denote the values (for a given STA-based parameter) for the baseline models, while the corresponding red dots represent the same for the VKMs. The parameter under consideration is mentioned below each sub-panel. **(a–e)** are for the CaN knockout, **(f–j)** are for the CaR knockout, while **(k–o)** are for the CaL knockout.

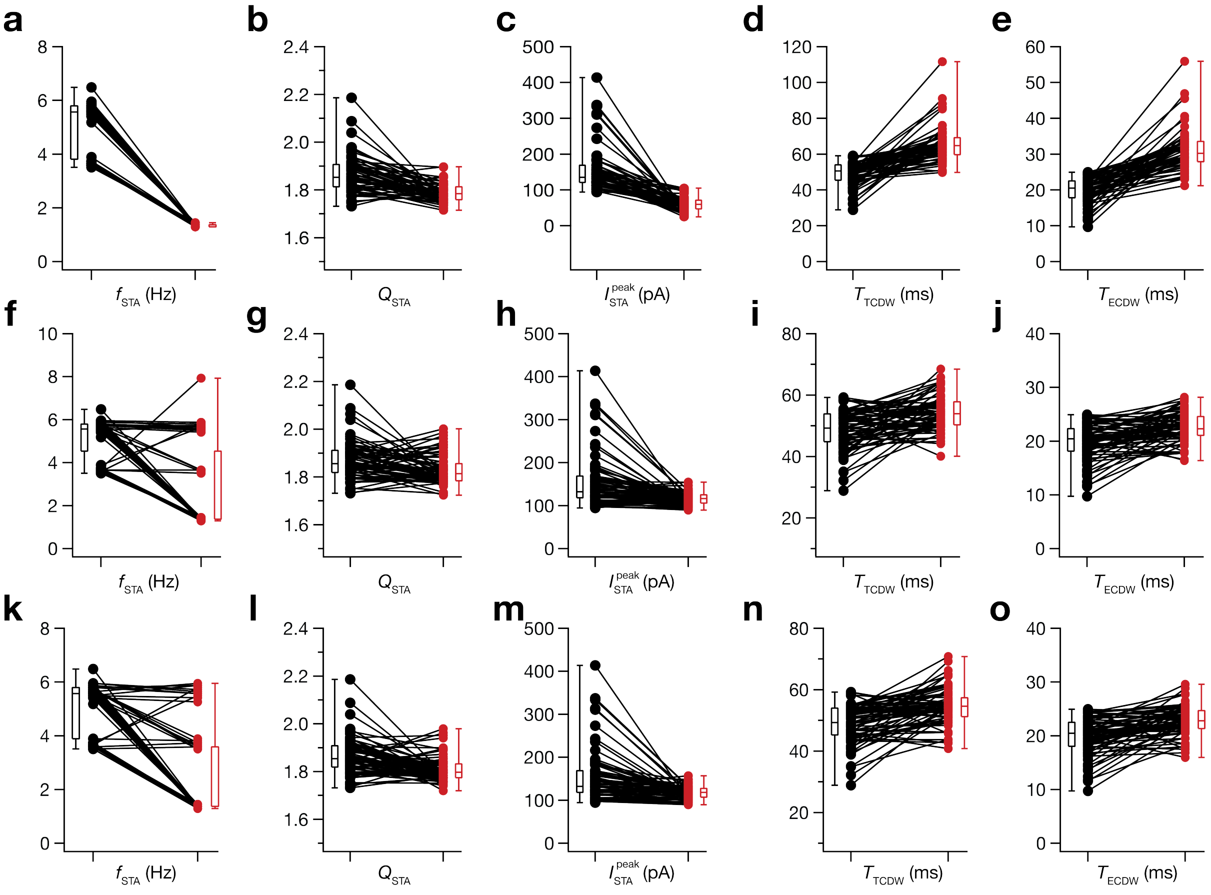


**Supplementary Figure S4:** Comparison of the five STA-based properties between the baseline models and the VKMs. In each subpanel, the black dots denote the values (for a given STA-based parameter) for the baseline models, while the corresponding red dots represent the same for the VKMs. The parameter under consideration is mentioned below each sub-panel. **(a–e)** are for the KA knockout, **(f–j)** are for the SK knockout, while **(k–o)** are for the BK knockout.

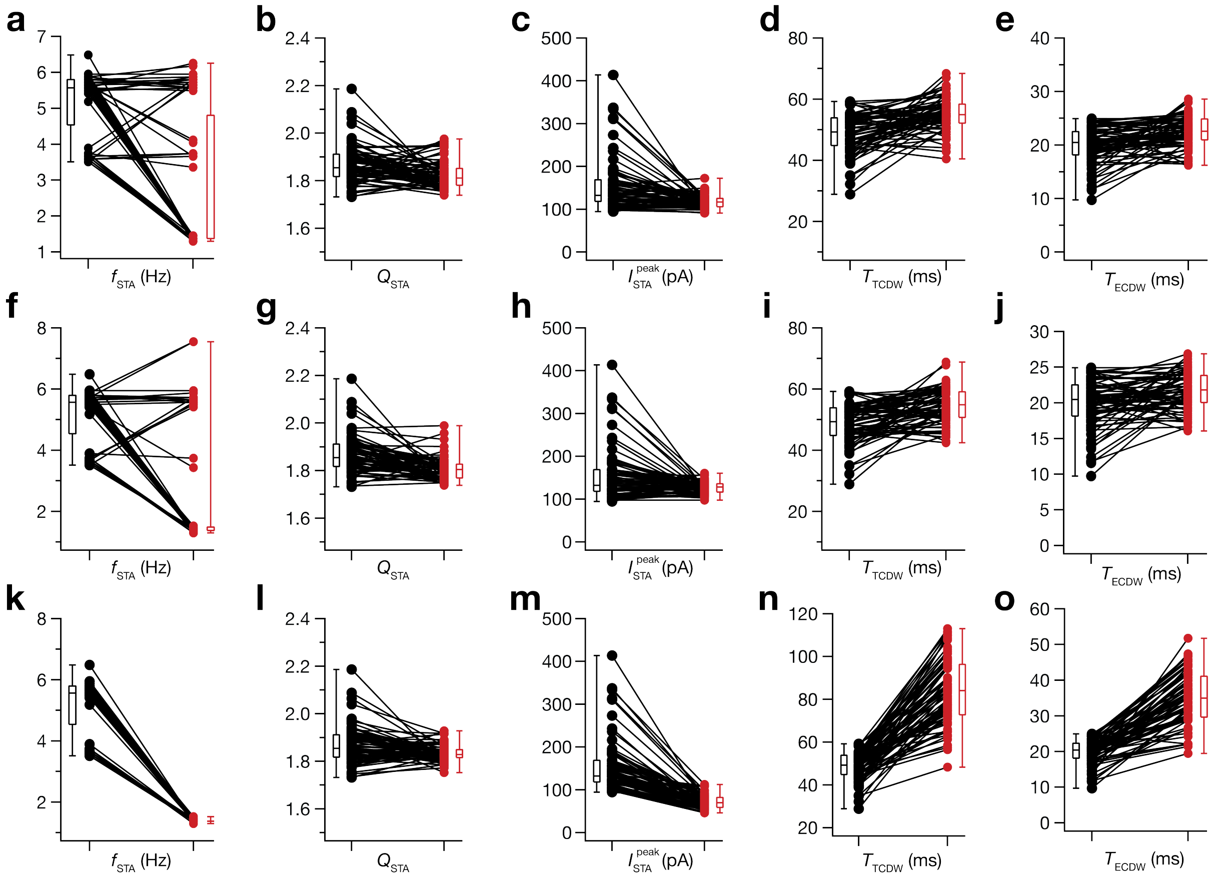


**Supplementary Figure S5:** Comparison of the five STA-based properties between the baseline models and the VKMs. In each subpanel, the black dots denote the values (for a given STA-based parameter) for the baseline models, while the corresponding red dots represent the same for the VKMs. The parameter under consideration is mentioned below each sub-panel. **(a–e)** are for the KM knockout, **(f–j)** are for the CaT knockout, while **(k–o)** are for the HCN knockout.
